# Electrically driven deep ultraviolet MgZnO lasers at room temperature

**DOI:** 10.1038/s41598-017-02791-0

**Published:** 2017-06-01

**Authors:** Mohammad Suja, Sunayna Binte Bashar, Bishwajit Debnath, Longxing Su, Wenhao Shi, Roger Lake, Jianlin Liu

**Affiliations:** 1 Department of Electrical and Computer Engineering, University of California, Riverside, CA 92521 United States; 2 School of Physics and Engineering, Sun Yat-sen University, Guangzhou, 510275 People’s Republic of China

## Abstract

Semiconductor lasers in the deep ultraviolet (UV) range have numerous potential applications ranging from water purification and medical diagnosis to high-density data storage and flexible displays. Nevertheless, very little success was achieved in the realization of electrically driven deep UV semiconductor lasers to date. In this paper, we report the fabrication and characterization of deep UV MgZnO semiconductor lasers. These lasers are operated with continuous current mode at room temperature and the shortest wavelength reaches 284 nm. The wide bandgap MgZnO thin films with various Mg mole fractions were grown on *c*-sapphire substrate using radio-frequency plasma assisted molecular beam epitaxy. Metal-semiconductor-metal (MSM) random laser devices were fabricated using lithography and metallization processes. Besides the demonstration of scalable emission wavelength, very low threshold current densities of 29~33 A/cm^2^ are achieved. Numerical modeling reveals that impact ionization process is responsible for the generation of hole carriers in the MgZnO MSM devices. The interaction of electrons and holes leads to radiative excitonic recombination and subsequent coherent random lasing.

## Introduction

Efficient laser devices operating in the deep UV region have a wide range of applications, which include fluorescence detection, water sterilization and purification, high-density data storage, medical diagnosis, and chemical and biological agent sensing^1–6^. Owing to high efficiency, scalable size, and low power consumption, semiconductor lasers have advantages over traditional inefficient frequency converted solid-state lasers or excimer UV lasers that require the use of hazardous materials^2^. UV spectral bands are commonly categorized into UV-AI (400~340 nm), UV-AII (340~315 nm), UV-B (315~280 nm), and UV-C (280~100 nm)^7, 8^. Over the last few decades, tremendous success was achieved on the development of electrically injected semiconductor laser diodes operating in the UV-AI band using GaN and ZnO based materials and their heterostructures^9–25^. In contrast, very little has been succeeded on electrically pumped laser diodes in the UV-AII and shorter bands. The only available reports to date include AlGaN based quantum well lasers having lasing peaks at ~336 nm^26^, MgZnO based metal-insulator-semiconductor (MIS) laser with lasing peaks at ~330 nm^27^, and AlGaN nanowire random lasers operating in the UV-AII, UV-B and UV-C bands^28–31^. Among all these nanowire-based lasers, only one has reached the shortest emission wavelength of 239 nm^31^. Various issues associated with wide bandgap AlGaN materials including ineffective p-type doping and large effective masses of both electrons and holes have contributed to the severe gap in deep UV laser development^30^.

Owing to the direct bandgap (3.37 eV) and high exciton binding energy (60 meV), ZnO has been studied extensively to realize low-threshold excitonic laser devices at room temperature^15–25, 32–50^. Wurtzite MgZnO ternary alloy is a direct, wider bandgap semiconductor that can lead to functional deep UV optoelectronic devices. Nevertheless, reliable p-type doping of ZnO and MgZnO has been extremely difficult. Thus a great deal of effort has been spent on the realization of lasers by circumventing the need for p-ZnO or p-MgZnO, including heterojunctions^51, 52^, Schottky diodes^24^, and metal-semiconductor-metal junctions^23^. In this paper, we report plasma-assisted molecular beam epitaxial (MBE) growth and characterization of MgZnO thin films on *c*-sapphire substrate. The polycrystalline MgZnO thin films possess high-density randomly distributed columns with air gaps between them, which act as random laser medium. Metal-semiconductor-metal (MSM) devices have been fabricated by depositing Au/Ti and Au/Ni Schottky contacts onto MgZnO films. We assessed several possible mechanisms and reached a finding that holes are generated near the space charge region of the reverse-biased metal-semiconductor junction during the operation of MgZnO MSM devices using a non-local impact ionization model in device simulation. A two-dimensional (2D) frequency domain mode analysis was performed to depict random lasing mode generation in the multiple grain structures. With this study, we have demonstrated electrically injected MgZnO based random lasers operating in the UV-B and UV-AII bands, pushing the shortest wavelength ever reported for any electrically injected semiconductor thin film lasers operating at room temperature from ~330 nm^26, 27^ down to 284 nm.

## Results

**Figure 1:**
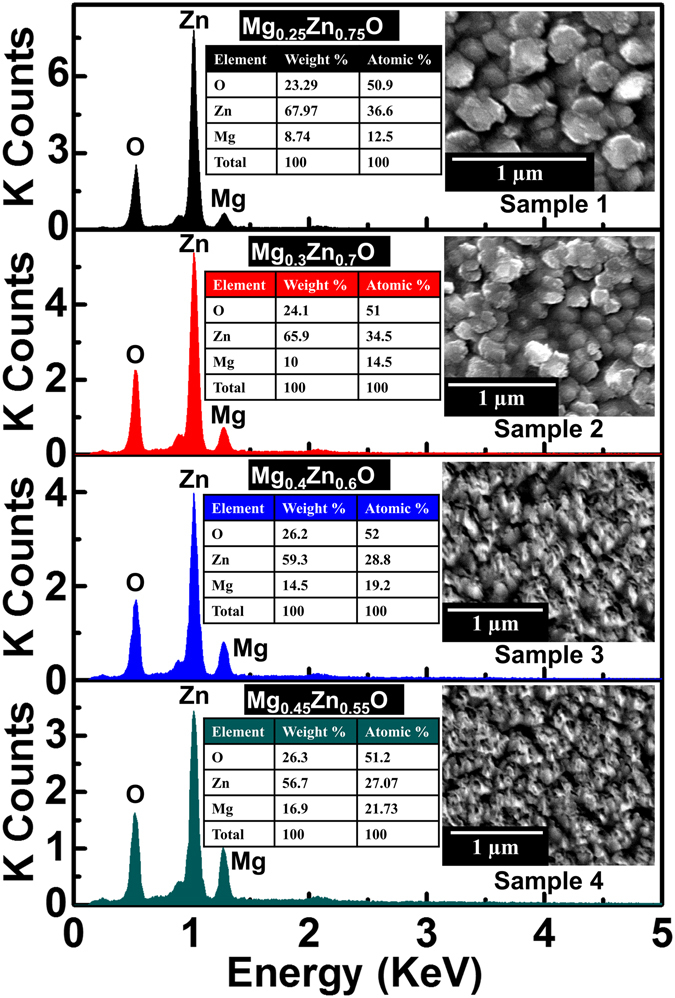
Composition and morphology of MgZnO thin films. EDX spectra of (**a**) Sample 1 (Mg_0.25_Zn_0.75_O), (**b**) Sample 2 (Mg_0.3_Zn_0.7_O), (**c**) Sample 3 (Mg_0.4_Zn_0.6_O), and (**d**) Sample 4 (Mg_0.45_Zn_0.55_O). Inset shows SEM images and tables summarizing the elemental compositions of the samples.

The details regarding growth conditions of MgZnO thin films (Samples 1~4) are summarized in the Methods section. Figure 1 shows energy dispersive X-ray (EDX) spectra of the MgZnO samples, displaying peaks corresponding to O, Zn, and Mg. From the EDX spectra, the Mg composition *x* in Mg_*x*_Zn_1−*x*_O can be calculated as 0.25, 0.3, 0.4 and 0.45 for Samples 1~4, respectively. Thus, the Mg concentration increases monotonically with the Mg effusion cell temperature. The inset in Fig. 1 shows SEM images of the samples. The presence of randomly distributed multiple grains and air gaps in the films is evident. The grain sizes for all samples are in the range of 130~300 nm. This kind of morphology originates from the low-temperature growth of the active layers on a large lattice mismatched sapphire substrate and facilitates effective light scattering for the formation of random laser cavities in the MgZnO active layers.

**Figure 2:**
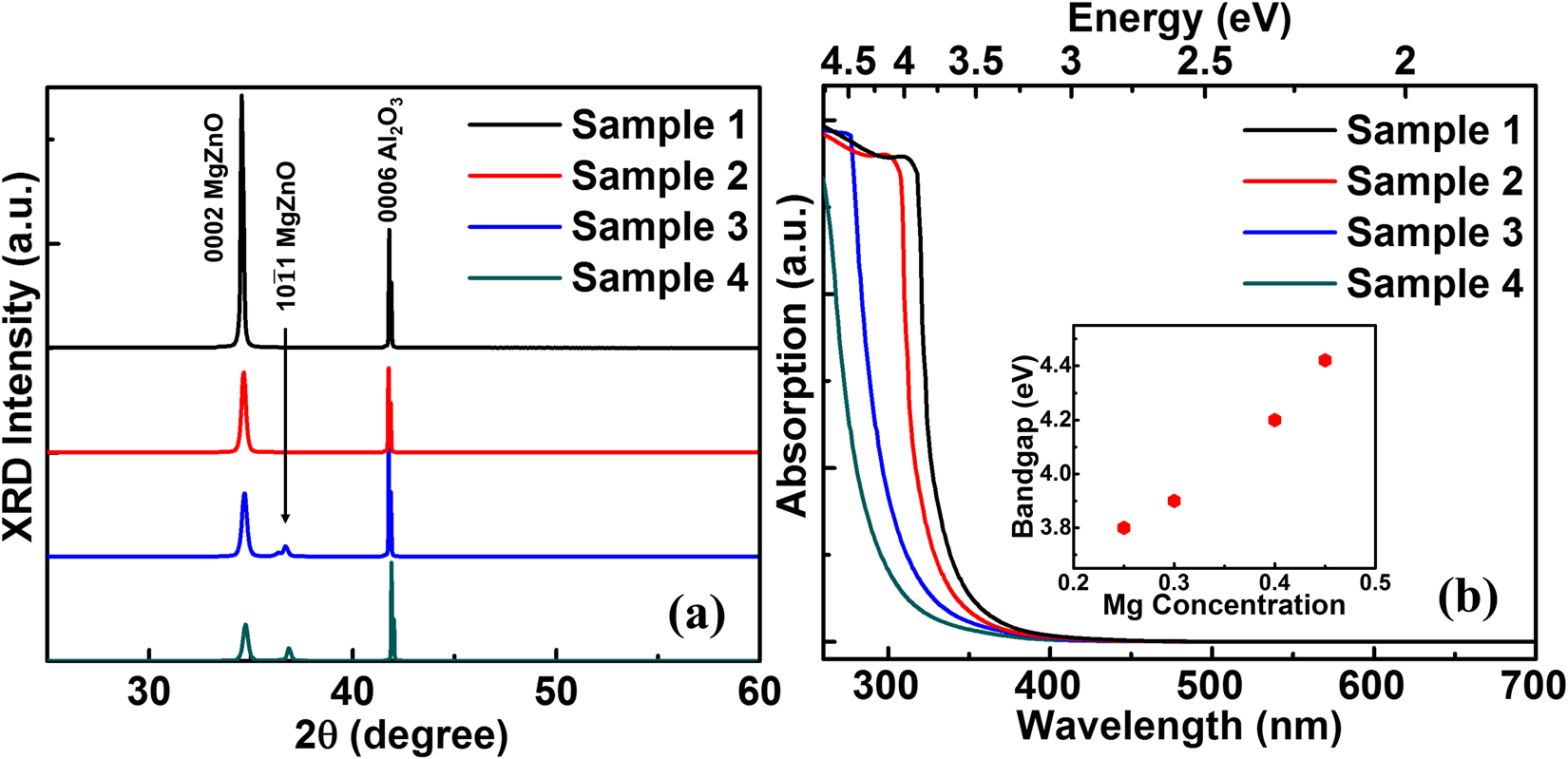
Structural and bandgap properties of MgZnO thin films. (**a**) XRD spectra of MgZnO thin films (Samples 1–4). (**b**) Room-temperature absorption spectra of MgZnO thin films (Samples 1–4), inset shows the variation of bandgap with Mg content in the film.

Figure 2(a) shows X-ray diffraction (XRD) spectra of Samples 1~4, respectively. The spectra exhibit diffraction peaks of MgZnO (0002) peaks and Al_2_O_3_ (0006) for all samples. The XRD data indicates that MgZnO films have grown in highly *c*-axis oriented hexagonal wurtzite lattice structure on *c*-sapphire substrates. Along with MgZnO (0002) peak, Samples 3 and 4 show MgZnO ($10\bar{1}1$) diffraction peak indicating the inclusion of cubic rocksalt structure into the hexagonal wurtzite structure. Nevertheless, the rocksalt phase is much weaker than its wurtzite phase. For all samples, the MgZnO (0002) peak positions have shifted to higher angle from 34.55° to 34.72° proportionally to the Mg contents in the films. The shift is due to the decrease in the lattice constant in the c-axis growth direction as a result of the substitution of Zn^2+^ ions (0.6 Å) by smaller sized Mg^2+^ ions (0.57 Å) in the host lattice^53, 54^. The *c*-axis lattice constant for MgZnO (0002) can be calculated by using the relation *c* = 2*d* = *λ*/*sinθ*, where d is the plane spacing along the *c*-axis, θ is the diffraction angle, and *λ* = 1.54 Å is the wavelength of X-rays^55^. The lattice constant shows a decreasing trend from 5.1859 Å to 5.1613 Å with the increase of Mg content (Supplementary Table 1). The broadening of full width at half maximum (FWHM) with the concentration implies the change in crystallographic characteristics of the films.

Figure 2(b) shows room temperature absorption spectra of the Mg_*x*_Zn_1−*x*_O thin films with various Mg concentrations (*x* = 0.25, 0.3, 0.4 and 0.45). The single slope absorption edges ensure no significant phase mixing in the MgZnO films even with higher Mg mole fractions. This result is in agreement with the XRD data in Fig. 2(a). The spectra reveal that with the increase of Mg content, the band edge of MgZnO shifts to shorter wavelength (blue shift). Typically, pure ZnO film has a band edge at ~380 nm^56^. For Sample 1, the MgZnO band edge is ~330 nm at 25% Mg concentration. The band edge shifts to ~320 nm for Sample 2 at 30% Mg concentration, and reaches a value of ~295 nm and ~280 nm at 40% (Sample 3) and 45% (Sample 4) Mg mole fraction, respectively. Evident increase of absorption coefficient near the absorption edge can be observed from all samples. High exciton binding energy (~60 meV) of ZnO and alloys play important role in the observation of these near-band-edge exciton absorption peaks in the absorption spectra at room temperature. The existence of excitons in the films at room temperature justifies the excitonic lasing in our devices as is shown later. For evaluating the bandgap of MgZnO, we have employed the derived spectrum of α^2^ versus photon energy (hν) at room temperature, where α is the absorption coefficient (Supplementary Fig. 1). The bandgap energy, E_g_ of the films can be calculated by utilizing Tauc’s plot assuming *α* ^2^ ∝ (*hv*−*E*_g_) relationship^32, 57, 58^. The inset of Fig. 2(b) shows the variation of Mg_*x*_Zn_1−*x*_O bandgap as a function of Mg concentration *x*. The bandgap widens in the range from 3.8 eV to 4.42 eV by introducing Mg content *x* from 25% to 45% in the Mg_*x*_Zn_1−*x*_O film. These bandgap engineering results are in good accordance with the experimental observation of Ohtomo *et al*.^32^. We have also measured the photocurrent spectra of MgZnO samples to further demonstrate the bandgap associated with the samples (see Supplementary Section 4). In both absorption and photocurrent spectra, no absorption associated with deep-level transitions at longer wavelengths is observed, indicating high optical quality of the films.

**Figure 3:**
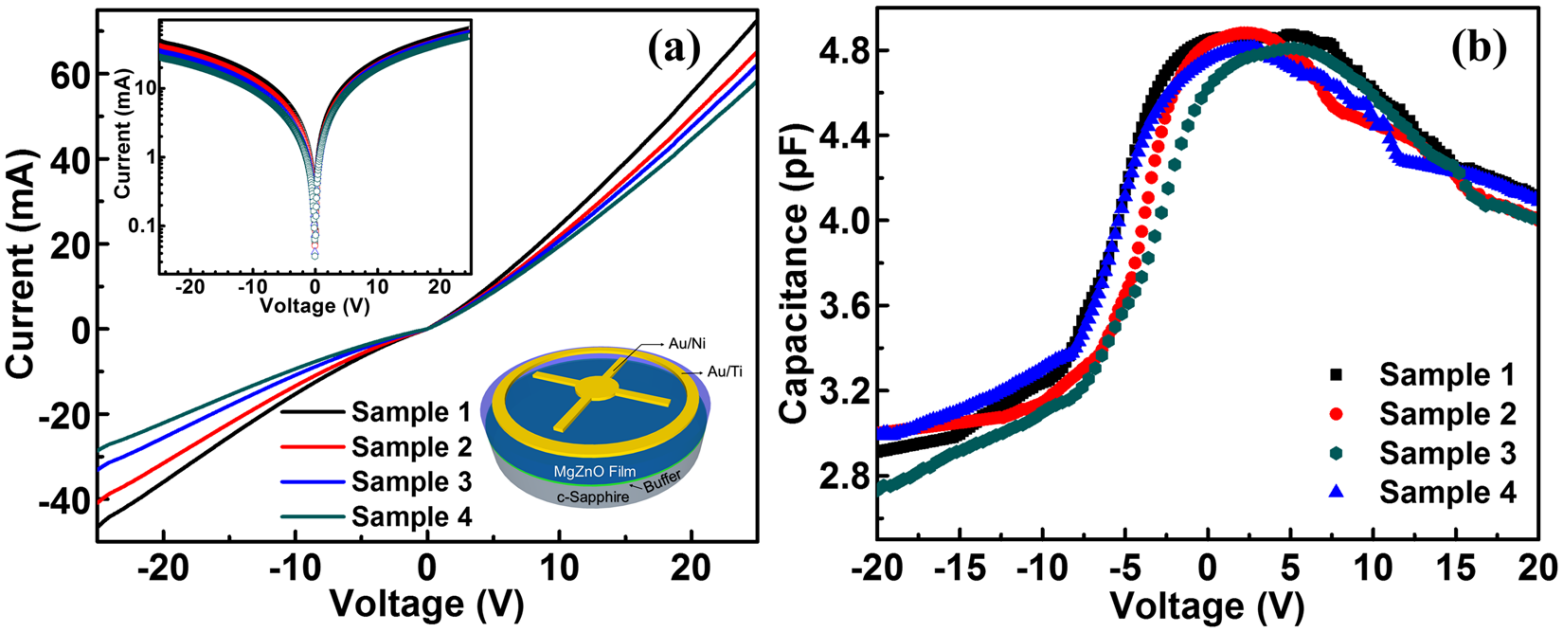
Electrical properties of MgZnO MSM devices. (**a**) I-V characteristics of Samples 1–4. Top left inset displays semi-log plot of I-V data. Bottom right inset shows a schematic of MSM device. (**b**) C-V characteristics of MgZnO MSM devices (Samples 1–4).

The electrical properties of the MgZnO films characterized by Hall effect measurement are summarized in Supplementary Table 2. These samples exhibit n-type conductivity with high resistivity (~10^2^ Ω-cm) and carrier concentration in the range of ~10^15^ cm^−3^. Moderate carrier mobility data is a reasonable result from the thin films consisting of packed grains. MgZnO MSM random laser devices have been fabricated and packaged using standard photolithography, contact metallization and wire-bonding techniques (see Methods). The bottom right inset of Fig. 3(a) shows a schematic of the fabricated devices. Au/Ni and Au/Ti metals act as the center crossbar and outer circular contact, respectively. The current-voltage characteristics are shown in Fig. 3(a). The top inset shows semi-log I-V of the MSM devices. Typical I-V characteristics of the MSM device, namely, two Schottky diodes placed back-to-back, are evident from the nearly symmetric saturation current behavior under both forward (designated as positive voltage on Au/Ni contact) and reverse biases^59, 60^. The monotonic decrease in current with increasing Mg concentrations suggests that MgZnO thin films with wider bandgaps are more resistive. Figure 3(b) shows capacitance-voltage (C-V) characteristics of Samples 1~4. The C-V curves depict usual behavior of an MSM device, namely, the capacitance of the devices decreases as the amplitude of both positive and negative voltages increases, which is mainly due to the increase of the space charge region width of the reverse-biased Schottky diode in the loop.

**Figure 4:**
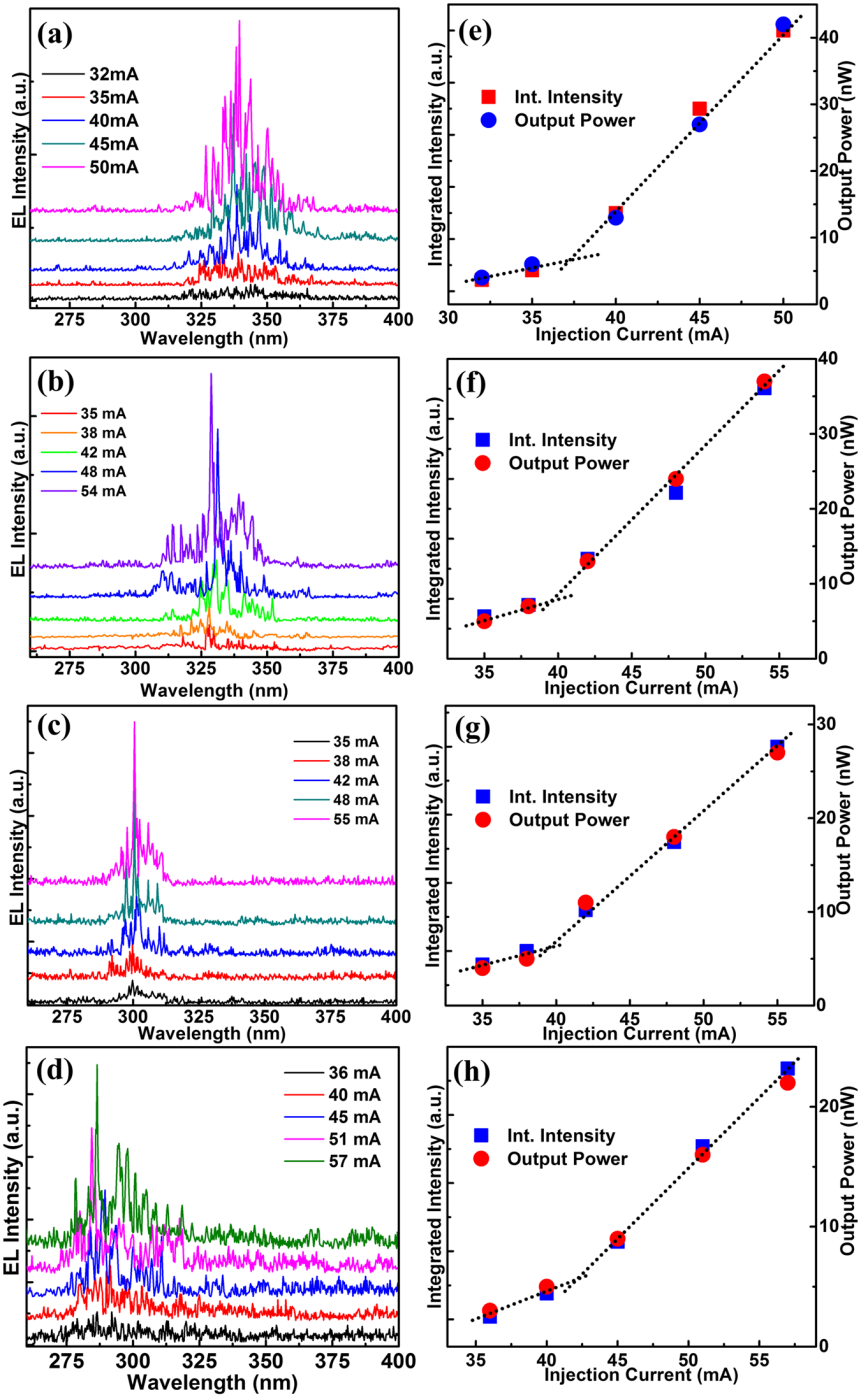
Electroluminescence and output characteristics of MgZnO MSM devices. (**a**–**d**) RT electroluminescence spectra of Samples 1–4 under different injection current. (**e**–**h**) Integrated intensity and output power as a function of injection current for Samples 1–4.

The lasing characteristics of MgZnO thin films were investigated by injecting continuous (dc) currents into the MSM devices (positive bias on center Au/Ni contact). All lasing spectra were collected from the top surfaces of the laser devices at room temperature. Figure 4(a–d) show electroluminescence (EL) spectra of Samples 1~4, respectively. All devices exhibit spontaneous emission at smaller injection currents, and stimulated emission superposing on spontaneous emission at higher injection currents. These sharp lasing modes with a FWHM of 0.5~0.7 nm are associated with the formation of closed-loop random laser cavities in these MgZnO thin films. The center lasing modes are clearly observed at 335, 325, 300, and 284 nm for Samples 1~4, respectively. To the best of our knowledge, the 284-nm emission is the shortest wavelength ever reported from any semiconductor laser device based on thin films under electrical injection at room temperature.

Figure 4(e–h) show the integrated EL spectral intensity and output power as a function of the injection current for the four devices, respectively. The output power was measured at the wavelength of the center emission in each device. The threshold behavior of the emission intensity and output power can be observed in all four plots. The threshold current is estimated to be ~37, 40, 40, and 43 mA for Samples 1~4, respectively. Considering the same device area of 1.26 × 10^–3^ cm^2^ for all devices, the threshold current densities of 29~33 A/cm^2^ are among the lowest threshold of lasers ever reported^26, 29, 30^. The output power of the center mode in the EL spectra is found to be about 40, 30, 20, and 15 nW at the same injection current of 50 mA for Samples 1~4, respectively. The smaller output power in the samples with higher Mg content may be related to the fact that it is more difficult to generate electron-hole pairs in the wider bandgap semiconductors.

The lasing action was demonstrated by applying positive bias not only at the center crossbar contact (Au/Ni) as shown in Fig. 4 but also at the outer circular ring contact (Au/Ti) (Supplementary Fig. 3, see Supplementary Section 5). Since the MSM device consists of back-to-back connected metal-semiconductor Schottky junctions, the occurrence of lasing emission under both voltage polarities suggests that these junctions are central element responsible for the lasing action, which is discussed later in detail. It should be noted that the EL spectra of Sample 2 in Supplementary Fig. 3 were measured three months after the first measurement. Although the intensity slightly decreases, obvious lasing emission can still be detected from the device under injection currents similar to original injection levels [Fig. 4(b)], suggesting reasonable reliability due to the fact that the devices were not passivated.

## Discussion

The observation of luminescence indicates the presence of radiative recombination of excitons. The number of excitons going through radiative recombination can be inferred from the output power of the emission if we tentatively do not include internal and external quantum efficiencies in the equation: ${P}_{out}=\frac{n}{t}\times h\nu$, where, *hv* is the photon energy. For example, since the measured output power for MgZnO MSM random laser device (Sample 4) is, P_*out*_ = 23*nW* at ~25 V [Fig. 4(h)], the number of excitons or roughly the number of holes per second (*n*/*t*) accountable for recombination is ~3 × 10^10^ cm^−3^/s. The total hole supply rate could be much higher than this crude estimation, considering the photons from both stimulated and spontaneous emissions at other wavelengths, and also fractional extraction and internal quantum efficiencies. Thus, it is necessary to elucidate the origin of the generation of holes in the unipolar MSM devices.

**Figure 5:**
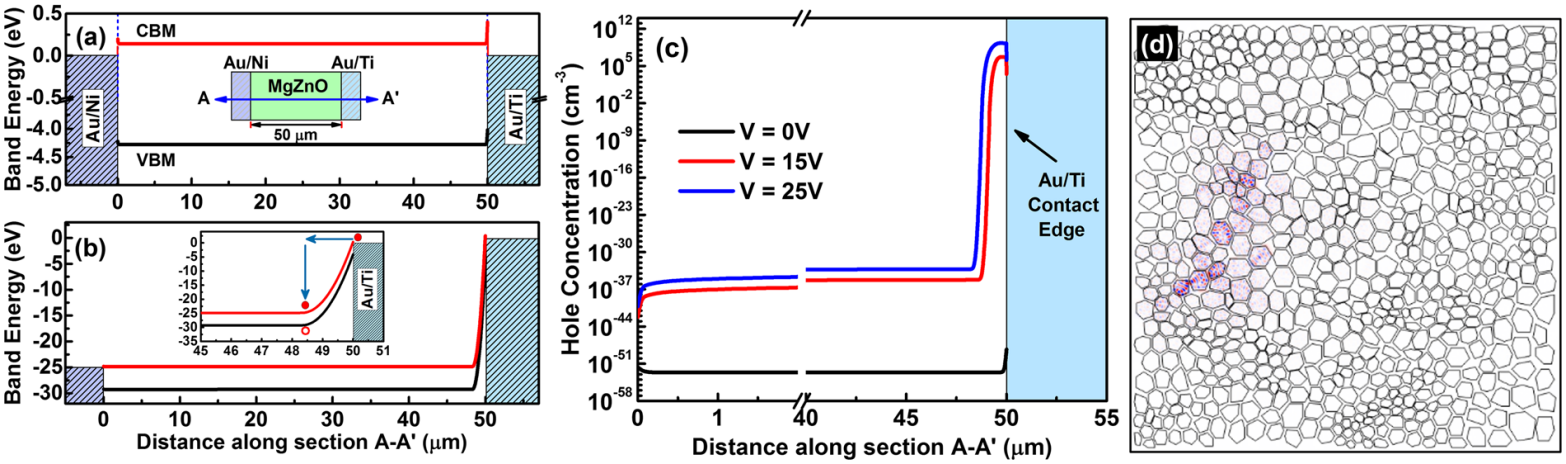
Simulation of hole generation and mode behavior in MgZnO MSM devices. Band diagram of MgZnO along A-A′ section direction for (**a**) V = 0 V and (**b**) V = 25 V. CBM and VBM in (**a**) stands for conduction band minimum and valance band maximum, respectively. The inset in (**b**) shows the impact ionization process by hot electrons in the high field region, (**c**) Hole concentration along A-A′ section from non-local impact ionization model, (**d**) The intensity of electric field distribution along c-axis corresponding to one possible lasing mode for Mg_0.45_Zn_0.55_O film (Sample 4).

The generation of the excess holes in the present MgZnO MSM devices assuming a positive voltage on the center Au/Ni contact could originate from several processes: hole leakage current from the forward-biased Au/Ni/MgZnO Schottky junction, trap-assisted band-to-band tunneling, and impact ionization near the reverse-biased Au/Ti/MgZnO Schottky junction. From the electrical properties, the n-type Au/Ni/MgZnO Schottky barrier is estimated to be on the order of a few tenths of an eV. Since the bandgap for the Mg_0.45_Zn_0.55_O film is 4.4 eV, thermionic emission of holes can be ignored. The 3D potential profile of the simulated device geometry shows that almost all of the potential drops in the space charge region near the reverse-biased junction (see Supplementary Section 6). Figure 5(a) and (b) show the band diagram along a narrow Mg_0.45_Zn_0.55_O channel region (~50 μm) between the inner Au/Ni contact fin and the outer Au/Ti circular contact (Sample 4) at equilibrium (0 V) and high bias (25 V) condition, respectively. The high field region corresponding to the depletion width of the reversed biased Au/Ti/MgZnO Schottky contact is ~1.5 μm. Due to the large 4.4 eV band gap and the relatively wide depletion region of the reverse-biased Au/Ti/MgZnO junction, the two-step trap-assisted tunneling process, modeled using Wentzel-Kramers-Brillouin (WKB) approximation, leads to negligible hole generation (see Supplementary Section 8). Several authors have explained the origin of holes, required for electroluminescence in ZnO devices, by the impact ionization process^61–63^. When the injected electrons from the Au/Ti contact enter the high-field space charge region, the carriers attain high energy before being scattered by optical phonons. The scattering length depends on the carrier saturation velocity and relaxation time. For ZnO, the reported hot electron energy relaxation time (*τ*) varies between 30 fs to 1.8 ps, depending on the growth condition, defects, and doping in ZnO^64^. In our MgZnO devices, we assume *τ* = 1.5 ps, which results in a scattering length of ~300 nm. The electrons gain excess energy over this distance and eventually excite excess electron-hole pairs by impact ionization. Figure 5(c) shows the estimated hole concentration from the non-local impact ionization model. The holes are mostly generated within the space charge region near the outer contact. The hole generation increases with applied bias. At a bias of 25 V, the highest steady-state excess hole concentration reaches 10^10^~10^11^ cm^−3^. This number is much smaller than the Mott density of 10^17^ cm^−3^ or so in ZnO^65^, which further suggests that the present lasing is excitonic-like rather than electron-hole plasma type. The resulting excitons, formed by the interaction of these hole carriers and abundant electrons flowing in the devices, paves the way to excitonic emission^66^.

The MgZnO films consisting of grains and air gaps act as highly scattered media to create a closed-loop cavity by multiple-scattering of photons as a result of excitonic recombination, which is a requirement of the realization of “coherent” random lasing^36^. Multiphysics COMSOL simulations were carried out to investigate possible random lasing modes in the present devices (see Methods). Figure 5(d) shows the simulated electric field distribution of a typical confined mode in the MgZnO random cavity under an operating wavelength of 280 nm (Sample 4). Multiple modes exist in the same cavity and the results are simulated and shown for all samples in the supplementary material section (see Supplementary Section 7). Multiple possible confined modes found in the different regions of the same cavity are consistent with the picture of closed light paths and the generation of random emission peaks detected in the EL measurements.

In summary, we have demonstrated room temperature electrically driven deep UV random lasers in the wavelength range down to 284 nm based on MBE grown MgZnO thin film MSM device structures. The hole generation in the MgZnO MSM devices is explained by impact ionization process through a comprehensive device modeling. Under the present conditions of operation of the devices, the number of excess holes is far less than Mott density, suggesting that the lasing is not in the region of electron-hole plasma. The excitons, formed through the interaction between these excess holes and electrons, can recombine radiatively, and with an assistance of a mode analysis, the resultant light is found to undergo multiple scattering and form closed-loop random cavities inside the multiple grain MgZnO structures, leading to extremely low threshold excitonic lasing. Although the output powers of the devices are relatively low at present due to limited supply of excess hole carriers, one way to increase the power is to enhance impact ionization process by applying higher voltage across the devices. Thus, this work suggests an effective way in the development of UV semiconductor lasers at around 280 nm. With further increase of Mg mole fraction in MgZnO alloy and effective control of their Wurtzite/rocksalt phases, it is possible to achieve deeper UV lasers.

## Methods

### MgZnO Thin Film Growth

MgZnO thin films were grown on one-inch diameter *c*-sapphire substrates by SVT associates (SVTA) molecular beam epitaxy (MBE) system equipped with a radio frequency (RF) plasma assisted oxygen source. High-purity elemental Zn (6 N) and Mg (6 N) were evaporated by using Knudsen effusion cells and the Zn and Mg fluxes were controlled by the effusion cell temperature. High-purity O_2_ (6 N) gas flow controlled by a mass flow controller was directed to the RF plasma source to generate active oxygen radicals. Prior to the growth, the sapphire substrate was cleaned in aqua regia (HCl:HNO_3_) solution at a temperature of 150 °C for 40 minutes, rinsed in de-ionized (DI) water, blown dry by nitrogen gas and transferred immediately to the MBE chamber. To achieve an atomically clean surface, the substrate was annealed in vacuum at 800 °C for 15 minutes. The growth procedure followed several steps. First, a MgO/ZnO buffer layer was deposited on the substrate at a substrate temperature of 450 °C for 5 minutes for all samples. The MgO/ZnO thickness in the buffer layer is varied from ~4.5/10.5, ~5/10, ~5.5/9.5, and ~6 nm/9 nm for Samples 1~4. The equivalent mole fraction of each element in the buffers was designed to be equal to that in the MgZnO active layers grown on top. During the buffer layer growth, the effusion cell temperature for Mg and Zn was kept at 450 °C and 305 °C, respectively, while O_2_ was introduced through the RF plasma tube at a flow rate of 1.5 sccm for all samples. The RF plasma power of 400 W was used during this step and MgZnO growth periods for all samples. In the next step, the samples were annealed at 650 °C for 5 minutes under ambient O_2_ with an O_2_ flow rate of 2.5 sccm. The active MgZnO layers were deposited at a substrate temperature of 350 °C, a Zn cell temperature of 300 °C and an O_2_ flow rate of 2.5 sccm. The Mg cell temperature was varied from 417, 420, 425, to 427 °C for Samples 1~4, respectively. The growth duration for the MgZnO active layers was 4 hours for all samples. At the final step, the samples were annealed *in-situ* at 700 °C for 20 minutes under ambient O_2_ with an O_2_ flow rate of 2.5 sccm. All samples yield a total thickness of ~650 nm.

### Structural and Optical Characterization

The thickness of the films was measured by a Veeco Dektek 8 profilometer system. X-ray diffraction (XRD) spectra were measured using a Bruker D8 Advance X-ray diffractometer. Scanning electron microscope (SEM) images were taken using a Philips XL-30 SEM machine. The SEM is equipped with an Energy Dispersive X-ray Spectrometer, which is utilized to measure energy dispersive X-ray (EDX) spectra. The experiments were performed using an electron beam with an acceleration voltage of 10 KV and a secondary electron detector. Room temperature absorption measurements were carried out by a Varian Cary 500 double-beam scanning ultraviolet/visible/near-infrared (UV/vis/NIR) spectrophotometer.

### Device Fabrication

For Hall effect measurement, Au/Ti (100 nm/10 nm) contacts were deposited on a Hall bar geometry sample by e-beam evaporation method and annealed at 400 °C for 1 minute by rapid thermal annealing process. MSM laser devices were fabricated by standard microfabrication process. The contacts comprise of an inner crossbar geometry with a circular ring (100 µm diameter) having four extended fins (100 µm × 20 µm) and an outer circular ring (outer diameter of 450 µm, inner diameter of 400 µm). Au/Ni (150 nm/20 nm) and Au/Ti (150 nm/20 nm) metals deposited by e-beam evaporation method were served as inner and outer contacts, respectively. The schematic of the devices is shown in the inset of Fig. 3(a). Finally, the fabricated devices were wire-bonded on TO5 cans by using a Hybond 572 A Wedge wire bonder and subsequently attached to a heat sink for electroluminescence (EL) measurements.

### Device Characterization

Hall effect measurement was carried out by a Keithley 6220 current source with a minimum current capability of 0.1 pA and up to 105 V compliance, and a Keithley 2182 voltage source with voltage capability of 1 nV. I-V characteristics were measured using an Agilent 4155 C semiconductor parameter analyzer. C-V characteristics were measured using an Agilent 4284 A LCR meter. Photocurrent was measured by using a 150-W Oriel Xe arc lamp as light source. The light from the lamp passed through an Oriel 0.25-m monochromator, which produced a specific wavelength light at its output port. After chopping, the light was then cast on the device. The generated photocurrent signal was fed to a lock-in amplifier where the data were collected. EL spectra were collected from the top surface of the wire-bonded device using an Oriel monochromator, a photomultiplier detector, and a lock-in amplifier. Currents were injected to the device using an external HP E3630A dc power supply. A heat sink was attached to the back side of the devices to control the temperature during the EL characterization. A Thorlabs PM100 optical power meter was used to measure the output power from the devices.

### Device Simulation

MgZnO MSM devices are simulated using both the finite element simulation tool COMSOL Multiphysics^67^ and the TCAD simulator Atlas^68^. The MgZnO channel is assumed to have an electron carrier concentration of 9 × 10^15^ cm^−3^ to be consistent with the experimental results. The effect of surface trap states and grain boundary defects are ignored. The bulk *c*-sapphire (Al_2_O_3_) substrate below the channel is modeled as an insulating boundary condition. Au/Ni/MgZnO and Au/Ti/MgZnO metal-semiconductor contacts are modeled as n-type Schottky diodes due to the difference of their work functions and electron affinity of MgZnO^23^. The Schottky barrier height used in the calculations is 0.4 eV. The electron mobility is 38 cm^2^/V·s, whereas the hole mobility is negligible^69^. The COMSOL model uses Fermi-Dirac statistics for both majority (electron) and minority (hole) carriers. The coupled drift-diffusion, carrier continuity, Poisson, and thermionic emission equations are solved numerically using a non-linear, iterative scheme. However, non-local effects, such as impact ionization and hot electron effects, which rely on thermal energy exchange between the electron (hole) and lattice, requires us to go beyond the traditional semiconductor model^70^. We use energy balance transport equations, as implemented in Atlas, which calculates carrier temperature from the linearized Boltzmann transport equation, assuming a Maxwellian shape to the distribution of hot carriers^71^. Subsequently, the calculated carrier temperature distribution is used as an effective non-local field (E_eff_) that describes the impact ionization model^72^. The electric field dependence of the impact generation coefficient in MgZnO is absent in literature. Hence, we take the impact generation coefficient of ZnO as a reasonable approximation for MgZnO: ${\alpha }_{MgZnO}=A\,\exp [-(\frac{B}{{E}_{eff}})]$, where *A* = 7 × 10^5^ *cm* ^−1^, *B* = 5 × 10^6^ *V*/*cm* are the calculated parameters of wide-gap ZnO^73^. The saturation velocity for electron in MgZnO sample is ~2 × 10^7^ cm/s^74^ and the relaxation time is assumed to be 1.5 ps^64^.

The frequency domain mode analysis using the radio frequency (RF) module of COMSOL Multiphysics 5.2 was used to simulate the electric field distribution in the MgZnO thin films with columnar structures. Given that the grain size and emission wavelength are smaller than the film thickness, a 2D simulation was adopted to investigate the in-plane lasing modes only. Randomly shaped and distributed hexagonal grains with lateral sizes of 200~400 nm and a filling factor of approximate 90% were generated. The morphology is closely in line with that of the devices studied here. The simulation area is chosen as 8 × 8 µm^2^ although similar results can be obtained with larger dimensions, which require much prolonged computer simulation time.

## Electronic supplementary material

Supplementary information
