# Supplementary material for: Electrically driven deep ultraviolet MgZnO lasers at room temperature
Source: Sci Rep. 2017 Jun 1;7:2677. doi: 10.1038/s41598-017-02791-0 (PMC5453953; doi:10.1038/s41598-017-02791-0)
Supplement: Supplementary file 1 — Supplementary information [file 41598_2017_2791_MOESM1_ESM.pdf]

Supplementary Information

Electrically driven deep ultraviolet MgZnO lasers at room temperature

Mohammad Suja [1], Sunayna Binte Bashar [1], Bishwajit Debnath [1], Longxing Su [1,2], Wenhao Shi [1], Roger Lake [1], and Jianlin Liu [1,*]

[1]*Department of Electrical and Computer Engineering, University of California, Riverside, CA 92521, United States*

[2]*School of Physics and Engineering, Sun Yat-sen University, Guangzhou 510275, People's Republic of China*

*Corresponding e-mail address: jianlin@ece.ucr.edu

Table of content

1. **Summary of XRD data of MgZnO samples**
2. **Electrical properties of MgZnO samples by RT Hall effect measurements**
3. **Tauc's plots of MgZnO samples**
4. **Photocurrent spectra of MgZnO samples**
5. **Lasing spectra at both forward and reverse biases**
6. **Electrostatic potential profile of MgZnO MSM device**
7. **Random lasing mode analysis of MgZnO MSM devices**
8. **Estimation of hole concentration from trap-assisted tunneling**

References

1. Summary of XRD data of MgZnO samples

The MgZnO (0002) peak shift with respect to the various Mg content in the samples are shown in Table S1. As the Mg content increases, the lattice constant decreases. The FWHM of the MgZnO (0002) peak broadens as the Mg content increases.

Table S1. XRD data of $Mg_xZn_{1-x}O$ Samples 1~4.

Samples (composition)	2θ (degree)	*c* (Å)	FWHM (degree)
Sample 1 ($Mg_{0.25}Zn_{0.75}O$)	34.55	5.1859	0.15
Sample 2 ($Mg_{0.3}Zn_{0.7}O$)	34.62	5.1757	0.23
Sample 3 ($Mg_{0.4}Zn_{0.6}O$)	34.68	5.1671	0.26
Sample 4 ($Mg_{0.45}Zn_{0.55}O$)	34.72	5.1613	0.27

2. Electrical properties of MgZnO samples by RT Hall effect measurements

Electrical properties of the MgZnO films are summarized in Table S2. All samples show a carrier concentration in the range of $1\sim4\times10^{15}$ cm^{-3}, mobility of 17~73 cm^2 V^{-1} s^{-1} and resistivity of 66~128 Ω-cm.

Table S2. Results of RT Hall effect measurements of MgZnO Samples 1~4.

Samples	Carrier Concentration (cm^{-3})	Mobility (cm^2 V^{-1} s^{-1})	Resistivity (Ω·cm)	Types
Sample 1	4.34×10^{15}	21.56	66.7	n-type
Sample 2	1.28×10^{15}	73.18	66.7	n-type
Sample 3	2.79×10^{15}	17.44	128.2	n-type
Sample 4	3.64×10^{15}	38.44	44.14	n-type

3. Tauc's plots of MgZnO samples

Figure S1 shows the derived Tauc's plots, namely, α^2 versus photon energy (hυ) of Samples 1~4 at room temperature, where α is the absorption coefficient. A direct band gap of 3.8, 3.92, 4.25 and 4.42 eV is obtained for Samples 1~4, respectively, by taking the intercept of the extrapolation to the zero absorption.

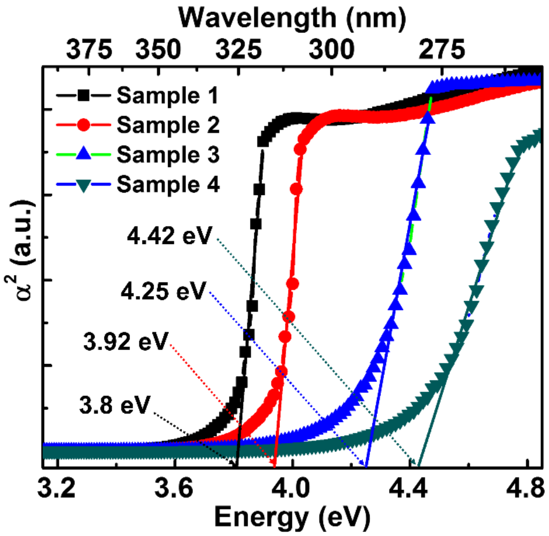

Figure S1. Square of absorption coefficient (α^2) at room temperature of Samples 1~4.

4. Photocurrent spectra of MgZnO devices

Photoresponse characterization was performed on the MgZnO MSM devices. Figure S2 shows photocurrent (PC) spectra under zero and forward (positive voltage on the Au/Ni contact) biases

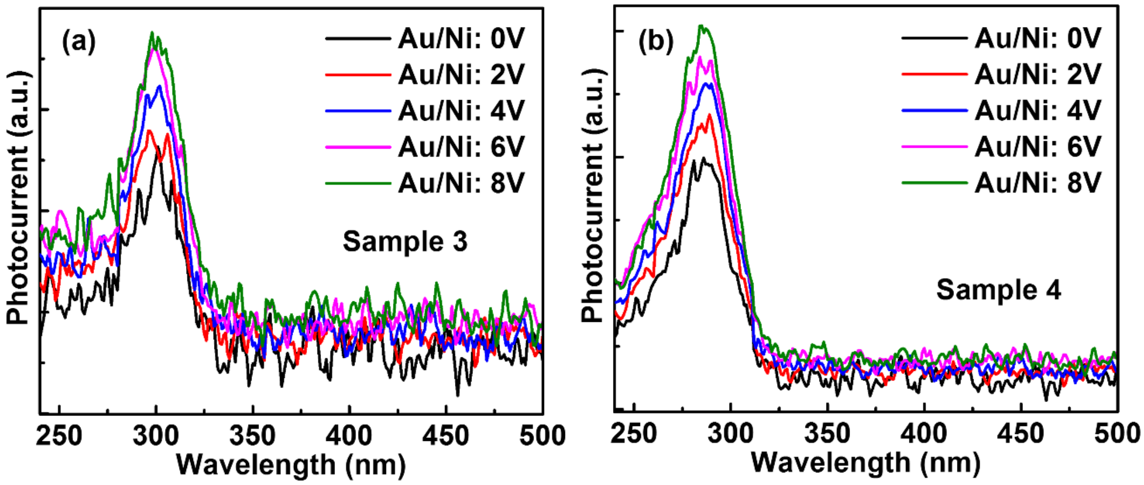

Figure S2. Photocurrent spectra under different biases (positive voltage on Au/Ni contact) of (a) Sample 3 and (b) Sample 4, respectively.

for the two representative samples (samples 3 and 4). The spectra show a peak at around 300 nm and 280 nm as the carriers are generated within the $Mg_{0.4}Zn_{0.6}O$ and $Mg_{0.45}Zn_{0.55}O$ layers,

respectively, and collected by the contacts. The photocurrent increases with the bias, which is due to generation and collection of more carriers under these conditions.

5. Lasing spectra at both forward and reverse biases

Figure S3 shows lasing characteristics of the MgZnO MSM device Sample 2 measured after three months from the first EL characterization by injecting similar currents into the device. Figure S3(a) shows the EL spectra when positive bias is applied on the Au/Ni contact. A broad spontaneous emission at around 325 nm can be observed under an injection current of 36 mA. After the current is increased to 45 mA, some sharp peaks begin to appear on the broad emission band. Further increase of the injection current to 60 mA leads to more sharp lasing peaks. The FWHM of these sharp peaks is about 0.6 nm and the peak in the center of the band is at ~325 nm. Figure S3(b) shows EL spectra of Sample 2 when the positive bias is applied on the Au/Ti contact.

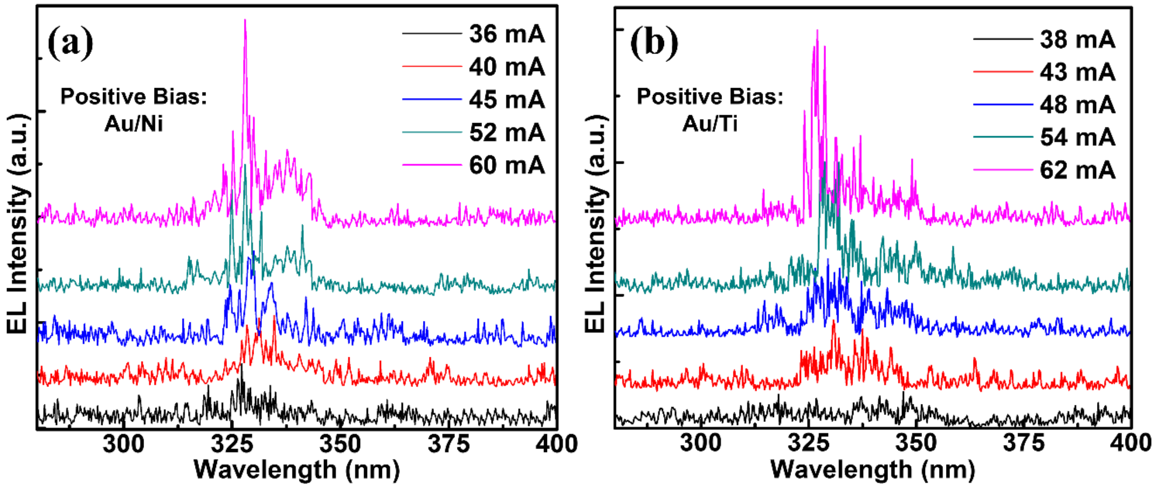

Figure S3. RT electroluminescence spectra of MgZnO Sample 2 under different injection current measured after 3 months, when (a) positive bias is applied on the Au/Ni contact, and (b) positive bias is applied on the Au/Ti contact.

The EL spectra displays sharp lasing peaks centered around 325 nm with a FWHM of around 0.7

nm as the injection current is higher than 48 mA, which is corresponding to the formation of random laser cavity in the $Mg_{0.3}Zn_{0.7}O$ film.

6. Electrostatic potential profile of MgZnO MSM device

Figure S4(a) shows the COMSOL 3D numerical simulation of electrostatic potential profile for different bias. The inner crossbar contact (Au/Ni) and outer circular contact (Au/Ti) have the same dimension as that of the fabricated sample. Both metal contacts (enclosed by black lines) are modeled as Schottky contacts. Positive bias V is applied either on the middle electrode (Au/Ni/MgZnO contact) or on the outer circular electrode (Au/Ti/MgZnO contact), resulting in reverse-biased junction at the outer circular contact and middle contact, respectively. Figure S4(b) and (c) show the 2D potential distribution around the respective reverse-biased contact. It is

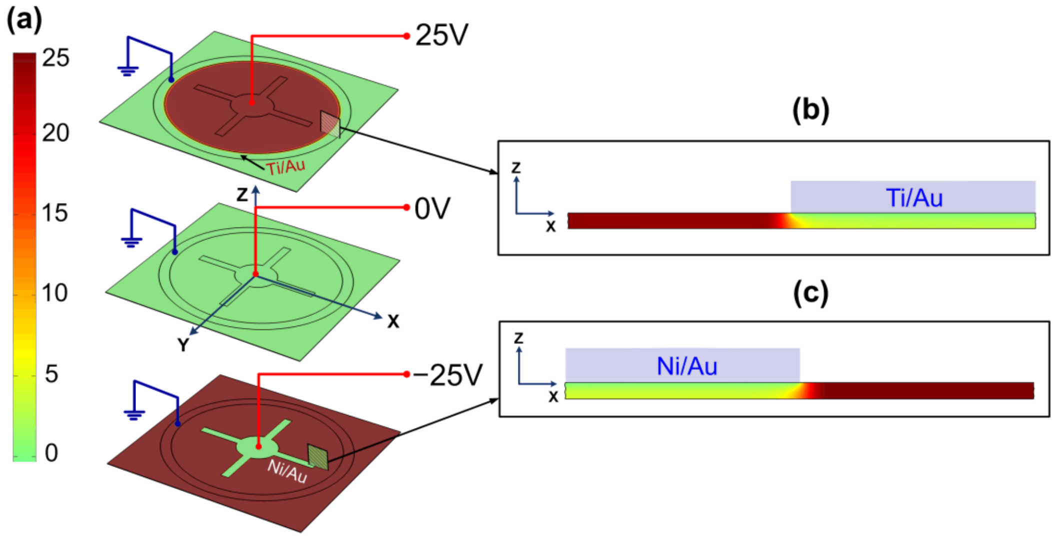

Figure S4. (a) electrostatic potential distribution over the simulated device structure, for V = 25V, 0V and -25V. The color scheme indicates the potential difference (ΔV) with respect to the lowest potential on the geometry. (b)-(c) are the 2D cross-section along X-Z and near the reverse biased Ti/Au and Ni/Au metal contacts, respectively. The color profile shows the sharp potential variation in MgZnO around the Schottky junctions.

evident that all the applied potential (25V) drops in the depletion region near the reverse-biased junctions.

7. Random lasing mode analysis of MgZnO MSM devices

To study the modes for all samples, the number of modes was set at six in the default settings of COMSOL to generate six modes that match the desired mode index. All the modes shown in the results have relative high possibilities to exist. From the optical properties of MgZnO material [1], mode effective index was set to be around 2, and the simulation results for the default six modes have a deviation to the reference mode index within 0.015%. Figures S5(a)–(d) show the simulated electric field distribution of six random confined modes under operating wavelength

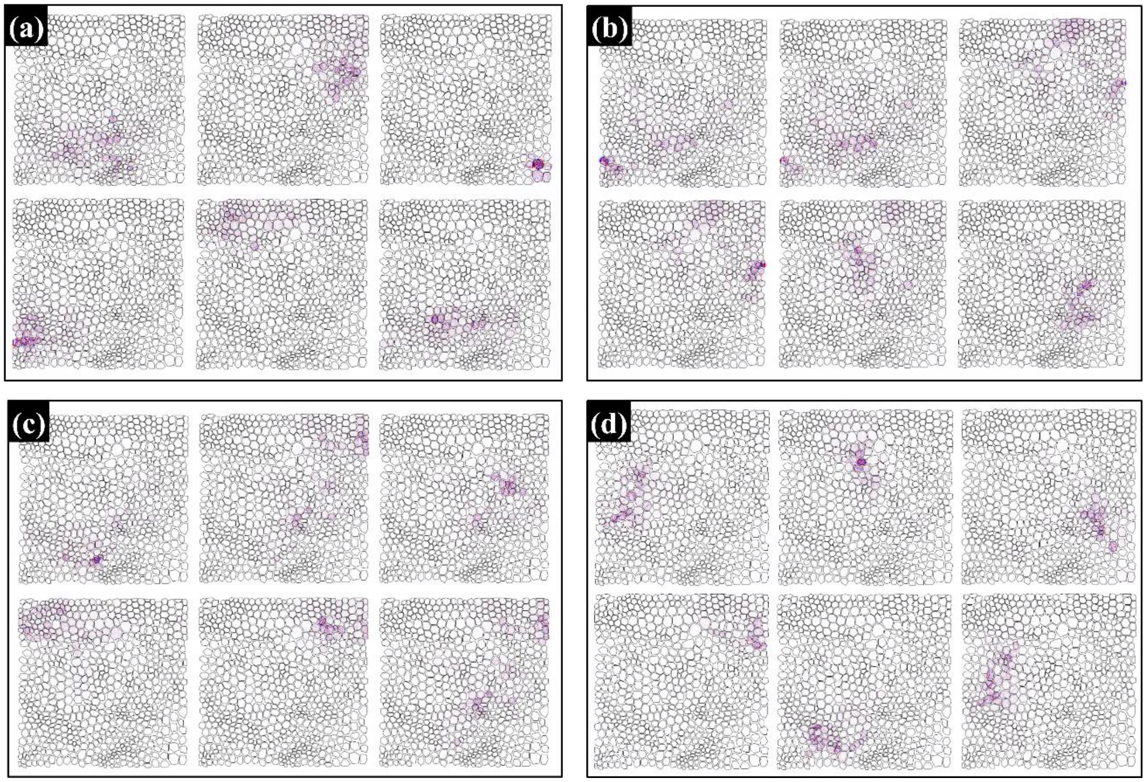

Figure S5. Mode analysis results of MgZnO samples with Mg composition of (a) 25%, (b) 30%, (c) 40% and (d) 45%. The operating wavelengths are 335 nm, 325 nm, 300 nm and 280 nm, respectively.

from 335 nm to 280 nm for the four MgZnO MSM devices (Samples 1~4), respectively. The Mg composition in the MgZnO film is varied from 25% for Sample 1, 30% for Sample 2, 40% for

Sample 3 and 45% for Sample 4 according to the experimental values. The dimension of the device area is chosen to be 8×8 μm^2. Several possible modes found in different regions of the film provide some correlated evidences for the multiple emission peaks detected in the EL measurements.

8. Estimation of hole concentration from trap-assisted tunneling

We have calculated the direct and trap assisted interband tunneling current as illustrated in Figure S5. For direct tunneling, the WKB approximation gives the tunneling probability from x_0 to x_2 as [3],

$$T_0 = \exp\left[-2\int_{x_0}^{x_2}|\kappa(x)|dx\right] = \exp\left[-\frac{2\varepsilon_I}{\varepsilon}\right], \quad (1)$$

where, ε is the electric field, $\varepsilon_I = \frac{1}{q}\int_0^{E_G} dE\ \kappa(E)$ is (1/q) times area under the E-κ curve where κ is the imaginary wavevector in the bandgap, $E = q\varepsilon x$ is the energy barrier, and ε is the electric field. Assuming a symmetric, parabolic, imaginary E-κ dispersion around $E_g/2$, $\varepsilon_I = \frac{2\sqrt{m^*}}{3q\hbar}E_G^{3/2} = 6.1\times10^7\ \left(\frac{\mathrm{V}}{\mathrm{cm}}\right)$, where $m^* = 0.3m_0$ is the effective mass, and $E_G = 4.4$ eV is band gap. Trap assisted tunneling (TAT), in the presence of a mid-gap trap-state, is a two-step tunneling process in which the electron tunnels from x_0 to x_1 with tunneling probability T_1 and then from x_1 to x_2 with tunneling probability T_2, as illustrated in Fig. S6. With a symmetric imaginary dispersion around midgap, these probalities are the same and given by

$$T_I = T_{II} = \exp\left[-2\int_{x_0}^{x_1}|\kappa(x)|dx\right] = \frac{1}{2}\exp\left[-\frac{2\sqrt{m^*}}{3q\hbar\varepsilon}\ E_G^{3/2}\right]. \quad (2)$$

Assuming the defect density N_T is sufficiently high such that $N_T\sigma_T > 1$, where σ_T is the capture cross section, then the trap assisted tunneling current is limited by the incident flux [6], so that the maximum TAT current is

$$J = \frac{2e}{hA}\sum_{k_t}\int dE\ T_{max}(E,\boldsymbol{k}_t)[f_{x_0}(E) - f_{x_1}(E)]$$

where $f_{x1}(E)$ is the occupation of the trap and $\boldsymbol{k}_t$ is the transverse momentum. For $T_I = T_{II}$, $f_{x1}(E)$=1/2. Since x_0 is in the valence band, $f_{x0}(E)$=1. Finite transverse momentum $\boldsymbol{k}_t$ corresponds to transverse energy $E_T = \hbar^2\boldsymbol{k}_t^2/2m^*$ which increases the effective bandgap to $E_G + 2E_T$. The total tunneling current density (J) is then

$$J = \frac{em^*}{2\pi\hbar^3}\int_0^{E_m} dE_T\ \int_{E_c+E_T}^{E_v-E_T} dE\ \exp\left[-\frac{2\sqrt{m^*}}{3q\hbar\varepsilon}\ (E_G + 2E_T)^{\frac{3}{2}}\right], \quad (3)$$

where, $E_m = (qV_A - E_g)/2$ is the maximum value of transverse energy (E_T) allowed. Performing the integral, the tunneling current is,

$$J = \frac{em^*}{\pi\hbar^3}\left(\frac{\varepsilon E_G}{3\varepsilon_I}\right)^2 e^{-\varepsilon_I/\varepsilon}\ \left[\frac{3\varepsilon_I}{2\varepsilon}\left(\frac{qV_A}{E_G} - 1\right) - 1 + \exp\left\{-\frac{3\varepsilon_I}{2\varepsilon}\left(\frac{qV_A}{E_G} - 1\right)\right\}\right], \quad (5)$$

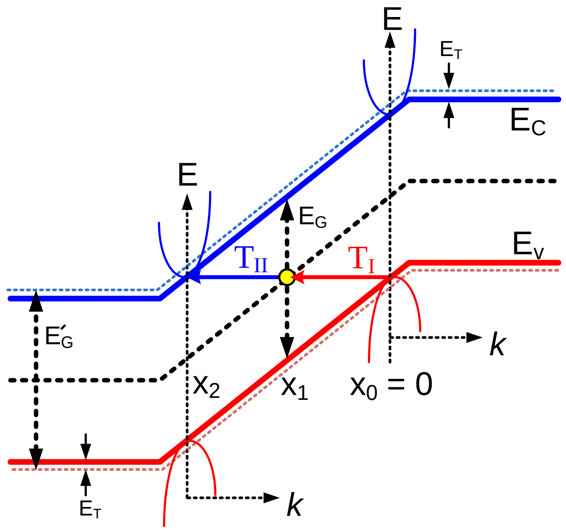

Figure S6. Schematic band diagram of trap-assisted tunneling from valance band (E_v) to conduction band (E_c). T_I denotes tunneling rate from valance band to deep-level trap-state, whereas T_{II} denotes subsequent tunneling from trap level to conduction band.

valid for $qV_A > E_G$. The order of magnitude is set by the factor $e^{-\varepsilon_I/\varepsilon}$. For a 25V bias and a 1μm depletion width, $\varepsilon = 2.5\times10^5\left(\frac{\mathrm{V}}{\mathrm{cm}}\right)$, and $e^{-\varepsilon_I/\varepsilon} = e^{-244} = 10^{-106}$. Thus, there is no hole generation from trap assisted tunneling.

References

[1] Teng, C. W. et al. Refractive indices and absorption coefficients of $Mg_xZn_{1-x}O$ alloys. *Appl. Phys. Lett.* **76,** 979 (2000).

[2] Kane, E. O. Zener Tunneling in Semiconductors. *J. Phys. Chem. Solids.* **12,** 181-188 (1959).

[3] Sze, S. M. & Ng, K. K. Physics of Semiconductor Devices. **5th ed** (Chapter 8), John Wiley and Sons, Inc, 2007.

[4] Parker, G. H. & Mead, C. A. The Effect of Trapping States on Tunneling in Metal-Semiconductor Junctions. *Appl. Phys. Lett.* **14,** 21 (1969).

[5] Gadzuk, J. W. Resonance Tunneling Through Impurity States in Metal-Insulator-Metal Junctions. *J. Appl. Phys.* **41,** 286 (1970).

[6] Suzuki, E., Schroder, D. K. & Hayashi, Y. Carrier conduction in ultrathin nitrided oxide films. *Appl. Phys. Lett.* **60,** 3616 (1986).
